# A cluster of invasive meningococcal disease (IMD) caused by *Neisseria meningitidis* serogroup W among university students, France, February to May 2017

**DOI:** 10.2807/1560-7917.ES.2017.22.28.30574

**Published:** 2017-07-13

**Authors:** Clément Bassi, Muhamed-Kheir Taha, Christian Merle, Eva Hong, Daniel Lévy-Bruhl, Anne-Sophie Barret, Ibrahim Mounchetrou Njoya

**Affiliations:** 1 Santé publique France, French National Public Health Agency, Regional Unit (Cire) Ile-de-France, Paris, France; 2 National Reference Centre for Meningococci and Haemophilus influenzae, Institut Pasteur, Paris, France; 3 Regional Health Agency in the Ile-de-France region (Agence régionale de santé d’Ile-de-France), Paris, France; 4 Santé publique France, French National Public Health Agency, Saint-Maurice, France; 5 These authors contributed equally to this work.

**Keywords:** cluster, invasive meningococcal disease, serogroup W, *Neisseria meningitidis*, university, MenACWY conjugate vaccine, South American/UK strain

## Abstract

Between February and May 2017, two cases of invasive meningococcal disease caused by a new, rapidly expanding serogroup W meningococci variant were reported among students of an international university in Paris. Bacteriological investigations showed that isolates shared identical genotypic formula (W:P1.5,2:F1–1:cc11) and belonged to the South American/UK lineage. A vaccination campaign was organised that aimed at preventing new cases linked to potential persistence of the circulation of the bacteria in the students.

In France, an increase of invasive meningococcal disease (IMD) due to the emergence of a new and rapidly expanding variant of *Neisseria meningitidis* serogroup W (South American/UK (UK) strain) has been observed since 2015 by the French National Public Health Agency and the French National Reference Centre for Meningococci (NRCM) in Paris (data not shown).

We describe here the detection of two cases, including one death, of laboratory-confirmed serogroup W IMD in university students in Paris between February and May 2017 and the rapid implementation of a vaccination campaign in the student community.

## Case reports and measures for contacts

### Case 1

At the end of February 2017, a student in his late teens (Case 1) was admitted to a hospital in Paris with signs of septic shock and *purpura fulminans*. At the admission, the body temperature was 37 °C. Symptoms started the day before the admission and the case had no underlying disease. The case, who lived and studied in Paris, was admitted to the intensive care unit and was immediately treated with cefotaxime. The student died the day following hospital admission and the health agency in the Ile-the-France region was informed the same day. An IMD was suspected at the admission, based on the clinical symptoms, and *Neisseria meningitidis* serogroup W was identified in a blood culture 6 days after the admission.

The health agency in the Ile-de-France region performed contact tracing immediately upon notification. According to French guidelines, close contacts who should receive chemoprophylaxis are those who have been directly exposed to the patient’s secretions from the nose or the throat in the 10 days preceding hospital admission of the case, typically household contacts or intimate friends. Vaccination is recommended around cases due to serogroups A, C, W or Y for contacts of the case if they are to remain in the case’s environment, even if they don’t have any close contact reported with the case as defined in the French guidelines [1].

Applying the guidelines, for 10 close contacts, post-exposure chemoprophylaxis with rifampicin was recommended. Moreover, MenACWY conjugate vaccine was recommended to 17 individuals belonging to the close circle of friends of the case, including those who had a post-exposure chemoprophylaxis recommendation. Six of them received the vaccine.

### Case 2

In May 2017, less than 3 months after the notification of Case 1, the health agency in the Ile-de-France region received another notification of IMD for another student admitted 2 days earlier at a local hospital. The case (Case 2), a young adult in their early twenties without any underlying disease, was a student at the same university as Case 1 and had been admitted with high fever (39,9 °C) and signs of meningitis and abdominal pain but without sign of *purpura fulminans.* Symptoms started the day before the admission. Serogroup W was identified in a blood culture 2 days after the admission. Of note, Case 2 had been vaccinated in 2010 using a MenACWY conjugate vaccine as recommended in the case’s home country.

Again, the local health authorities performed contact tracing immediately and according to French guidelines, the administration of post-exposure chemoprophylaxis was recommended for 22 identified close contacts and a recommendation of vaccination was done for eight friends. Five of them had already been vaccinated earlier and three received the vaccine.

### Epidemiological investigation and international aspects

Epidemiological investigation revealed that Case 1 and Case 2 followed different courses at the university. They lived in private accommodation off campus and had different groups of friends. For Case 1, 10 close contacts were identified and 22 for case 2. No common close contact between both cases was identified.

The university is an international higher educational institution with about a thousand students of 105 nationalities. The university year finished around mid-May 2017 and most students went home for the holidays. A summer course takes place in June for ca 350 students (200 students already studying at the university and 150 new students).

### Molecular typing

Isolates from both cases were sent to the NRCM where genotyping was performed. The characterisation by whole genome sequencing (WGS) showed that the isolates shared identical genotypic formula (W:P1.5,2:F1–1:cc11). The WGS data were analysed using the PubMLST Genome Comparator tool SplitsTree4 (version 4.13.1) which is used to visualise the resulting distance matrices as Neighbour-net networks [2,3]. Both isolates belonged to the South American/UK (UK) lineage (Figure). The European isolates of this lineage are clustered into two sub-lineages: the 'original UK strain' and the novel variant 'the 2013-strain'. The two isolates in this report belonged to the 2013-strain [4,5]. Furthermore, they clustered with other isolates isolated in France which seems to represent a new variant of the 2013-strain (Figure).

**Figure:**
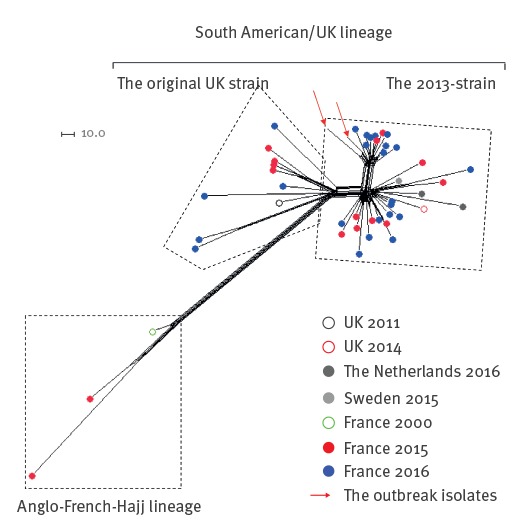
Phylogenetic network of group W/clonal complex (cc)11 isolates, France, 2015–2016 (n = 47)

UK: UK; WGS: whole genome sequencing.

WGS data are analysed using a gene-by-gene approach available through the PubMLST Genome Comparator tool. The nomenclature of lineages was according to [4,5].

The Anglo-French-Hajj and the South American/UK lineages are indicated as well as the sub-lineages: the 'original UK strain' and the novel variant 'the 2013-strain'. Five European isolates (from the UK, the Netherlands and Sweden) were included to represent these two sub-lineages as well as one French isolate from the Anglo-French-Hajj lineage. Data of the isolates are available through their PubMLST IDs (20196, 30167, 31165, 38691, 40279, 40387, 40391, 40399, 40401, 41212, 41582, 41728, 41812, 41819, 41857, 41864, 42046, 42202, 42206, 42209, 42213, 42409, 42758, 42767, 42769, 42782, 43996, 44053, 44434, 44573, 45339, 45341, 45342, 46742, 46743, 46744, 46745, 46746, 46747, 46748, 46749, 46750, 46751, 46752, 50353, 52131, 53526). The two recent French isolates are indicated by red arrows.

## Public health action

Following the occurrence of Case 2, the Regional public health agency convened a multidisciplinary expert group which decided to organise a vaccination campaign among the university students. The campaign using a MenACWY conjugate vaccine, aimed at preventing new cases linked to the potential persisting circulation of the bacterium in this community.

The target population were students staying in Paris during the holidays who would have social contacts with other university students in the weeks following the vaccination campaign, as well as those who were going to follow the summer course in June (ca 350 students). Students who had already returned to their home and with no planned activities at the university during the summer holidays were excluded from the recommendations, as were the educational and administrative staff of the university.

Two rounds of vaccination were organised between 1 and 9 June. A total of 186 students were vaccinated during the two rounds. Students who had received a dose of MenACWY conjugate vaccine more than 5 years ago were also included in the vaccination recommendation, taking into account the possibility of waning immunity following vaccination with a single dose of MenACWY conjugate vaccine.

## Discussion

During the 1990s, major increases in the incidence of IMD were observed in several western countries [6,7] with serogroup C ST-11 complex strain being the most noticeable and serogroup W being rare. Indeed, there were between three and 16 serogroup W isolates per year in France between 1994 and 1999 [8]. However, serogroup W of the clonal complex ST-11 (cc11) underwent a clonal expansion in France and other European countries in the year 2000, during the first multinational outbreak of serogroup W/cc11 which spread among pilgrims to Mecca and their contacts [9-11]. Since 2015, a new upsurge in serogroup W IMD has been observed in France in relation with the clonal expansion of the South American/UK strain [4]. This strain was first detected in South America [12], from where it later spread to the UK (UK) [13]. An increase in the incidence of serogroup W IMD was also reported recently in Spain [14], Australia [15] and the Netherlands [16].

We described here two cases of IMD caused by the serogroup W South American/UK strain, which occurred among students in a university in Paris within 3 months in 2017, without direct epidemiological link. This finding suggests a transmission through asymptomatic carriers at the university despite chemoprophylaxis recommendation to close contacts after the occurrence of the first case. It is expected that the targeted vaccination followed by the long summer holidays will interrupt the transmission of the strain before the beginning of the 2017/18 scholar year.

Given the time that has elapsed since the last case, students who left the university campus and are currently at home are not at risk for the disease, since it is generally thought that IMD occurs within 1 to 10 days after acquisition of an invasive strain [17]. Given the scattering of the students in their countries, students are not considered at particular risk of IMD if they do not frequent, in the short term, the university community where a higher risk has been identified. The risk of further transmission in their social group in their own countries seems low but cannot be ruled out.

The university setting and living conditions of students have probably contributed to the transmission in the cases described here since all first-year students in this university live in shared accommodation. Generally, it is known that university students have higher rates of IMD than young adults of the same age who are not attending university [18-20].

Interestingly, one of the two cases presented with gastrointestinal symptoms at admission. This presentation is atypical but seems to be not uncommon in cases infected with the South American/UK strain as reported in a case review in the UK [21]. Health professionals should thus be aware of this atypical presentation which can delay the diagnosis and appropriate treatment.

This is the second cluster episode linked to the South American/UK strain that occurred in a university setting in France since 2016. The first episode was in a university in another region (Burgundy region) and lead to a mass vaccination campaign targeting around 30,000 persons. In both clusters, isolates belonged to the novel variant, the 2013-strain. Furthermore, WGS analysis suggests that they may represent an expansion of a new local variant of the 2013-strain.

The strain appears highly transmissible and virulent. Association of the South American/UK lineage with outbreaks was suggested to be linked to a heightened carriage, transmission, invasiveness or virulence of this lineage [5]. Further studies will be helpful to understand these features in relation to the characteristics of the strain as well as the carriage rate and duration.
